# Latent Fingerprint Detection with SYPRO Rose Plus Protein Blot Stain

**DOI:** 10.1100/tsw.2002.133

**Published:** 2002-03-01

**Authors:** Kimberly K. Bouldin, E. Roland Menzel

**Affiliations:** Center for Forensic Studies, Texas Tech University, Lubbock, TX 79409, USA

**Keywords:** criminalistics, fingerprints, fluorescence, photoluminescence, lasers, time-resolved imaging, europium, lanthanide complexes, europium chelates, SYPRO^® ^ Rose

## Abstract

Lanthanide complexes are employed in photoluminescence detection of fingerprints because their long luminescence lifetimes allow use of time-resolved imaging techniques to suppress problematic background fluorescence. To date, however, these complexes have been unsuccessful when used in developing old fingerprints on porous substrates. SYPRO Rose Plus Protein Blot Stain remedies this shortcoming; it lends itself to smooth surfaces as well, thus having potential as a universal fingerprint reagent.

## INTRODUCTION

In principle, photoluminescence detection of fingerprints, especially when done with powerful lasers, provides unsurpassed sensitivity, given that today's technology has reached the single-photon detection regime. However, the print one wishes to detect is often located on an article that emits intense background fluorescence of a color similar to that of the fingerprint itself, so that optical filtering cannot be employed to suppress the background. This ubiquitous problem can be overcome by time-resolved imaging, a technique that allows one to effectively eliminate background fluorescence while retaining the luminescence from the fingerprint[1,2]. In time-resolved imaging, the photoluminescence lifetime of the fingerprint must be substantially longer than that of the background. Lanthanide complexes have the necessary long luminescence lifetimes and thus have been investigated for some time[2]. Current lanthanide-based techniques are effective in dusting and staining applications, namely development of fingerprints on smooth surfaces by physical processes. For instance, they are quite effective in concert with the cyanoacrylate fingerprint treatment, in much the same way as the postcyanoacrylate staining with fluorescent dyes such as rhodamine 6G[2]. The objective, however, is background fluorescence suppression, which traditional dyes do not provide, and wherein lies the truly essential sensitivity gain. Unfortunately, current lanthanide-based fingerprint treatments are generally not effective with prints older than a few days on porous articles, where chemical fingerprint development pertains. The most widely employed lanthanide complexes involve europium ($Eu^{3+}$), which, under ultraviolet excitation, luminesces at about 615 nm with a lifetime of millisecond order. It thus is a great candidate for time-resolved imaging. However, $Eu^{3+}$ itself absorbs poorly, hence will emit poorly. Its luminescence intensity is radically increased in chelates with ligands that absorb well and then transfer the excitation energy to the europium ion. This type of ligand is often referred to as a sensitizing ligand. For chemical fingerprint development, the europium complex also has a ligand that serves to chemically bind the complex to constituents of the latent fingerprint (conjugating ligand). $Eu^{3+}$ has coordination number 9. Thus, $Eu^{3+}$ in many complexes is not fully coordinated and the remaining coordination sites are taken up by waters of hydration. Unfortunately, water is a notorious quencher of lanthanide luminescence. Thus, completion of the $Eu^{3+}$ coordination via additional ligands to exclude waters of hydration is necessary to obtain the desired intense lanthanide luminescence. A ligand may serve both the conjugating and sensitizing functions, or it may be designed simply for coordination completion. At times, several sensitizing ligands are attached to the $Eu^{3+}$. A case in point is the mixed ligand europium complex reported by Lock et al.[3]. This complex does not have a conjugating ligand. It is thus designed for dusting and staining modalities. The sensitizing ligands, ortho-phenanthroline and thenoyl trifluoroacetone, broaden the range of effective excitation wavelengths, with ortho-phenanthroline responding to far UV and thenoyl trifluoroacetone responding to near UV excitation.

## SYPRO® Rose

SYPRO® Rose Plus Protein Blot Stain[4], obtained from Molecular Probes, is a europium complex with an organic component classified by Molecular Probes. Our efforts to obtain information on the complex from Molecular Probes were unsuccessful. The unreacted compound luminesces poorly, whereas the luminescence becomes intense upon binding to proteins. Thus, upon protein reaction, the $Eu^{3+}$ coordination goes from incomplete to complete, or a hydrophobic environment results from the reaction. Since Molecular Probes advertises SYPRO® Rose Plus Protein Blot Stain as a protein reagent, we speculate that it may attack amino (NH or $NH_2$) functional groups. Indeed, its application to amino acid (e.g., glycine) spots results in reaction concomitant with massive increase in luminescence at the spot site as compared to vicinal locations at which unreacted SYPRO® Rose resides. Application to spots that do not contain the $NH_2$ functionality, such as palmitic acid, results in no such luminescence gain. Given that we do not have access to the structure of SYPRO® Rose, we cannot, however, definitively establish the nature of the chemistry. Amino acid–based fingerprint development, as epitomized by the traditional ninhydrin treatment and the more recent diazafluoren(9)one (DFO)[2], has long been the workhorse of chemical fingerprint processing. SYPRO® Rose shows a broad absorption at wavelengths less than about 380 nm, peaked at about 350 nm, and the characteristic europium luminescence, of about 10-nm width, peaked at 615 nm. Upon reaction, the emission is so intense that illumination with an ordinary UV lamp is sufficient for viewing and photography purposes when background fluorescence is not an issue. In such instances, one obtains sensitivities roughly comparable to ninhydrin/zinc chloride[2] and DFO. Again, however, we emphasize that SYPRO® Rose is designed to be used in concert with time-resolved imaging to permit detection of fingerprints on items on which the traditional fluorescence-based techniques fail because of the excessive fluorescence background.

The SYPRO® Rose Plus protein kit, as received from Molecular Probes, is comprised of three components. Solution A serves to prewash membranes onto which proteins have been electroblotted. Solution B, which is an aqueous solution containing additives classified by Molecular Probes and the europium complex, serves the visualization of the proteins via the

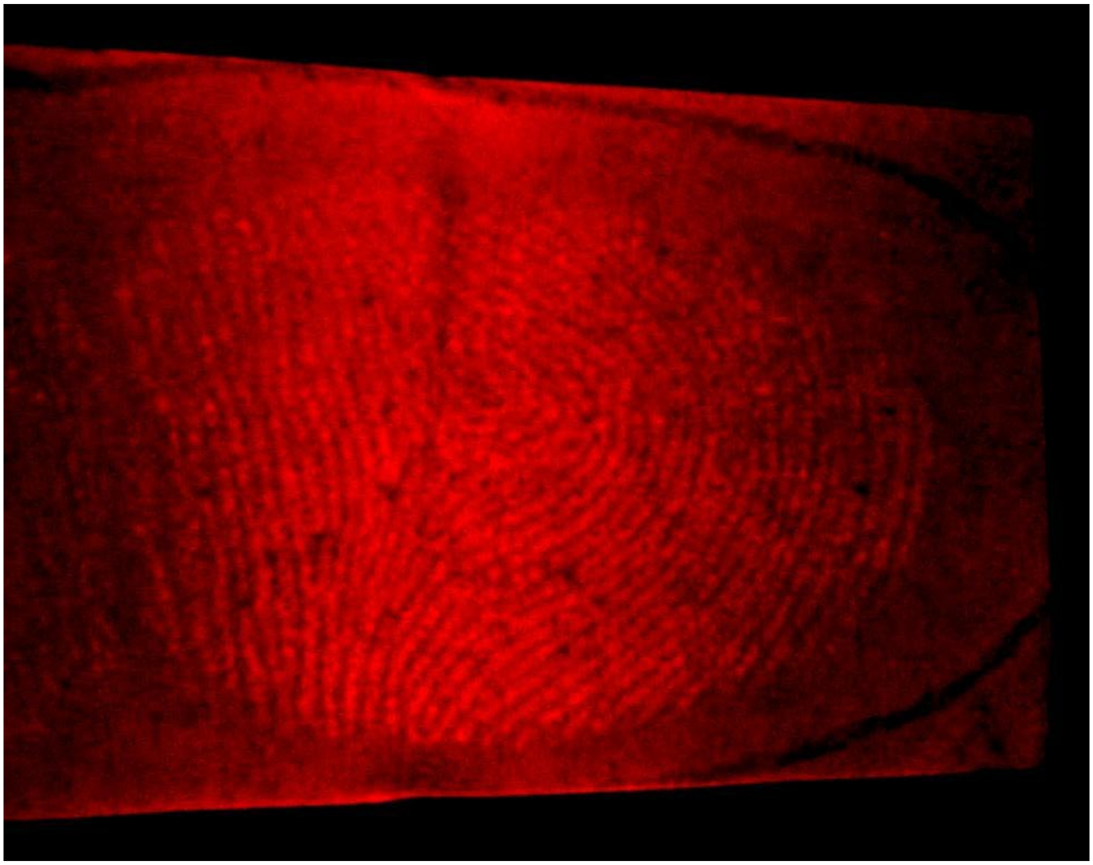

**FIGURE 1**. Three-month-old fingerprint on paper developed by SYPRO® Rose.

europium luminescence. Solution C serves to destain the proteins for further processing as needed. For fingerprint work, solutions A and C are not relevant. Only solution B is utilized. The Molecular Probes material safety data sheet on solution B does not list any known health hazard. The procedure for developing fingerprints using SYPRO® Rose is simple. The article holding the latent fingerprint is immersed in undiluted SYPRO® Rose solution B for about 24 h at ambient conditions. The article is then removed and left to dry. It is finally examined under UV excitation and viewed through a band-pass filter that transmits at 615 nm. If background fluorescence is severe, the article is subjected to time-resolved imaging. Clear fingerprint ridge detail is found for fingerprints on paper, even when such prints are older than 2 months. Fig. 1 depicts such an instance. As fingerprints on paper age, the relevant ingredients migrate into the paper, we suspect, rather than remaining on the surface, and thus become less accessible to chemical reaction, especially with large molecules, as europium complexes tend to be. Furthermore, incomplete coordination of the europium ion is detrimental to europium luminescence efficiency (waters of hydration mentioned earlier) as is weak chemical binding. Both problems have been encountered in past work with europium complexes. Apparently, SYPRO® Rose does not suffer from these problems. Indeed, the destaining solution C contains as its main ingredient ethylenediaminetetraacetic acid, which is an aggressive ligand. On occasion, we have been able to detect fingerprints on highly problematic porous substrates such as leather. SYPRO® Rose is also effective for detection of fingerprints on smooth surfaces generally, as shown in the instance of Fig. 2. The complex thus shows promise as a universal fingerprint detection reagent.

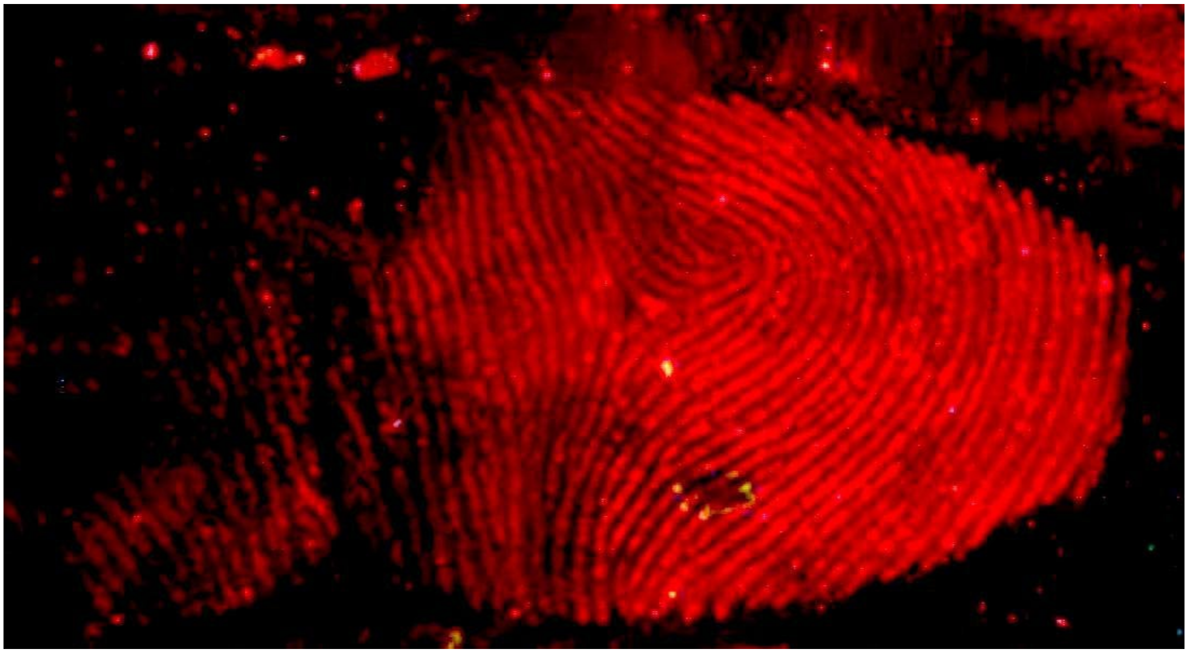

**FIGURE 2.** One-month-old fingerprint on aluminum foil developed by SYPRO® Rose.
